# The efficacy and safety of mitomycin C intra urethral injection to prevent recurrent urethral stricture: A systematic review and meta-analysis

**DOI:** 10.1016/j.amsu.2022.103576

**Published:** 2022-04-04

**Authors:** Firmantya Hadi Pranata, Furqan Hidayatullah, Yudhistira Pradnyan Kloping, Zakaria Aulia Rahman, Fikri Rizaldi, Doddy Moesbadianto Soebadi

**Affiliations:** a Department of Urology, Faculty of Medicine, Universitas Airlangga, Dr. Soetomo General-Academic Hospital, Indonesia; b Department of Urology, Faculty of Medicine, Universitas Airlangga, Universitas Airlangga Teaching Hospital, Indonesia

**Keywords:** Mitomycin C, Direct vision internal urethrotomy, Urethral stricture, Stricture recurrence

## Abstract

**Objectives:**

Direct Vision Internal Urethrotomy (DVIU) is regarded as the most popular and frequently used minimal invasive approach for treating urethral stricture. However, the application of this procedure is limited due to the high recurrence rate. Recent trials have the benefit of mitomycin C as adjuvant therapy to reduce the stricture recurrence in DVIU procedures. In this meta-analysis, we aim to determine the efficacy of mitomycin C as adjuvant therapy for DVIU.

**Methods:**

A systematic literature search was carried out from Embase, ScienceDirect, and PubMed published up to September 2021. Relevant Randomized Controlled Trials (RCTs) were screened using our eligibility criteria. The quality assessment of the RCT was assessed using Cochrane RoB 2. The outcome was measured as an Odds Ratio (OR) with 95% Confidence Intervals (CIs). Statistical analyses were performed using RevMan 5.4.

**Results:**

We included four RCTs in the meta-analysis, with a total of 392 patients with urethral strictures undergoing DVIU. The pooled analysis showed a significantly lower recurrence rate in patients undergoing DVIU with additional treatment of mitomycin C compared to the control group (OR 0.27, 95% CI 0.16–0.45, p < 0.0001).

**Conclusion:**

Our findings highlight the benefit of adjuvant treatment with mitomycin C to reduce the incidence of urethral stricture recurrence after DVIU procedures.

## Highlights

• Lower recurrence rate was observed in patients undergoing DVIU receiving mitomycin C.
• Mitomycin C could inhibit DNA synthesis thus repairing urethral lumen.
• Mitomycin C's anti-proliferative properties can suppress the growth of cancer cells and preventing recurrent urethral stricture.

## 1 Introduction

Urethral stricture is defined as a narrowing part of the urethra caused by fibrosis and scarring of the urethral mucosa and spongiosus tissue. The incidence of urethral stricture is reported to be around 229–627 per 100,000 population, which mostly affects the anterior portion of the urethra. Various factors have been associated with urethral stricture, including sexually transmitted infections, inflammation, external trauma, iatrogenic trauma, catheterization, and history of instrumentation or urethral surgery [1]. Urethral strictures might cause obstructive and irritating symptoms that impact the patients physically, mentally, and financially. In order to treat this disease, various procedures have been introduced, including the minimally invasive approach and reconstructive approach. Because of the high success rate and low recurrence rate, the reconstructive approach or urethroplasty is still considered the gold standard procedure in managing urethral stricture [2]. On the other hand, Direct Vision Internal Urethrotomy (DVIU) is regarded as the most popular and frequently used minimal invasive modality for treating urethral stricture [3]. However, the application of DVIU is limited because this procedure could only be performed on certain indications such as primary urethral stricture, single defects with <2 cm length, and non-obliterative in the bulbous urethra. In addition, the success rate of this procedure is reported to be only about 50%, and the recurrence rate of is ranged from 9%–60% [[4], [5], [6]]. Because of the high failure rate, repeated DVIU is not recommended [1]. Various studies have investigated the factors and interventions that could increase the success rate and lower the recurrence rate in DVIU. Recent studies have shown that neoadjuvant mitomycin C used in DVIU could significantly reduce the recurrence rate of urethral stricture after the DVIU [7]. Furthermore, Randomized Controlled Trials (RCTs) have been conducted to evaluate the effectiveness of this agent in reducing urethral stricture. However, the result from pooled analysis on the efficacy of mitomycin C in DVIU procedure has not been well-established. Therefore, in this systematic review and meta-analysis, we aimed to determine the efficacy of mitomycin C as adjuvant therapy for reducing the recurrence rate in DVIU procedures.

## 2 Methods

### 2.1 Study design

This systematic review is in accordance with the Preferred Reporting Items for Systematic Review and Meta-Analysis 2020 (PRISMA) Protocol [8]. AMSTAR-2 criteria was also implemented as the guidance to complete this review [9]. The protocol of this review has been registered in the PROSPERO registry (CRD42021249209) and research registry (reviewregistry1331)

### 2.2 Search strategy and eligibility criteria

All authors performed the electronic search from Embase, ScienceDirect, and PubMed search engines up to September 2021. Keywords related to mitomycin C and DVIU were searched. The full search strategy was summarized in Table 1. The inclusion criteria used in this review were: (1) RCTs comparing the use of mitomycin C and no mitomycin C after DVIU, (2) patients diagnosed with simple urethral strictures, (3) participants were men with age >18 years. We excluded studies that reported complex stricture populations, pediatric patients, and non-English articles.

**Table 1:** Search strategy used in pubmed.

Database	Keywords
Pubmed, Embase, ScienceDirect	((“dviu"[All Fields] OR ((“direct"[All Fields] OR “directed"[All Fields] OR “directing"[All Fields] OR “direction"[All Fields] OR “directional"[All Fields] OR “directions"[All Fields] OR “directivities"[All Fields] OR “directivity"[All Fields] OR “directs"[All Fields]) AND (“vision s"[All Fields] OR “vision, ocular"[MeSH Terms] OR (“vision"[All Fields] AND “ocular"[All Fields]) OR “ocular vision"[All Fields] OR “vision"[All Fields] OR “visions"[All Fields] OR “visioning"[All Fields]) AND (“internal"[All Fields] OR “internally"[All Fields] OR “internals"[All Fields]) AND (“urethrotomies"[All Fields] OR “urethrotomy"[All Fields])) OR (“urethrotomies"[All Fields] OR “urethrotomy"[All Fields]) OR (“mitomycin"[MeSH Terms] OR “mitomycin"[All Fields] OR “mitomycine"[All Fields] OR “mitomycins"[MeSH Terms] OR “mitomycins"[All Fields] OR (“mytomycin"[All Fields] AND ″c basel"[Journal]))) AND (“urethral stricture"[MeSH Terms] OR (“urethral"[All Fields] AND “stricture"[All Fields]) OR “urethral stricture"[All Fields])) AND (clinicaltrial[Filter])

### 2.3 Data collection and risk of bias assessment

Two independent reviewers performed the process of study selection and data collection. Any disagreement in deciding on eligible articles was resolved by discussing the full text with the senior authors. Data collection was performed using a piloted form consisting author name, year of publication, country, total sample, average age, recurrence rate, and follow-up duration. All these data were extracted and presented in the tabulation table. Each reviewer assessed the RCT risk of bias using the Cochrane Risk of Bias (RoB) 2 tool, which comprised of five domains [10]. The certainty of the evidence was assessed using a GRADE evidence profile.

### 2.4 Statistical analysis

To assess the heterogeneity among the studies, we used the I^2^ index and P-value. A random-effects model was applied if there was a high evidence of heterogeneity (P < 0.05, I^2^ > 50%). Otherwise, we analyzed the data using a fixed-effects model (P > 0.05, I^2^ < 50%). The primary outcome of this study was categorical data expressed as an odds ratio (OR) with a 95% confidence interval (95% CI). The forest plots containing the pooled analysis were generated using statistical software RevMan version 5.4 (Cochrane Collaboration, Oxford, UK).

## 3 Results

### 3.1 Study search and selection

From the multiple databases searching process, we identified a total of 117 records. After removing the duplicates, we screened 87 studies and assessed 17 full-text articles. Finally, we found four RCTs eligible to be included in the pooled analysis. The PRISMA guideline was implemented in the study selection. Several studies were excluded due to several reasons, including inappropriate study design [[11], [12], [13], [14]], inappropriate comparison [[15], [16], [17], [18]] [[15], [16], [17], [18]] [[15], [16], [17], [18]], observational studies [16,[19], [20], [21]] and an abstract-only articles [22].

### 3.2 Baseline characteristics of included studies

Table 2 summarizes the baseline characteristics of the included RCTs in our study. All of the included RCTs were performed in Asia, with a total of 392 urethral stricture patients undergoing DVIU procedures. The average age of the participants was 43 years old. All participants included in this study had urethral strictures for the first time. There was no significant difference in terms of length of the urethral stricture at the start of the treatment in each of the analyzed studies, with an average stricture length of 1.35 cm. The duration of follow-up of patients in this study differed in each trial. The follow-up period was ranged from 12 to 24 months.

**Table 2:** Baseline characteristics of the included study.

Author	Year	Design	Group	n	Mean age ± SD (years)	Mean stricture length ± SD (cm)	Recurrence rate (%)	Follow-up (months)
Islam et al.	2019	RCT	MMC	78	53.7 ± 24.75	1.07 ± 5.9	2/20	12
			PCB	78	54.5 ± 21.25	0.955 ± 4.15	10/20	
Mazdak et al.	2007	RCT	MMC	20	29.8 ± 14.8	0.76 ± 0.15	2/20	24
			PCB	20	29.2 ± 13.9	0.74 ± 0.17	10/20	
Ali et al.	2015	RCT	MMC	78	37.31 ± 10.1	1.86 ± 1.2	11/78	18
			PCB	73	40.1 ± 11.4	1.67 ± 1.4	27/78	
Yasser et al.	2021	RCT	MMC	21	31.2 ± 11.1	1.71 ± 1.2	3/21	12
			PCB	24	32.1 ± 11.3	1.63 ± 1.1	12/24	

### 3.3 Quality assessment

**Fig. 1:**
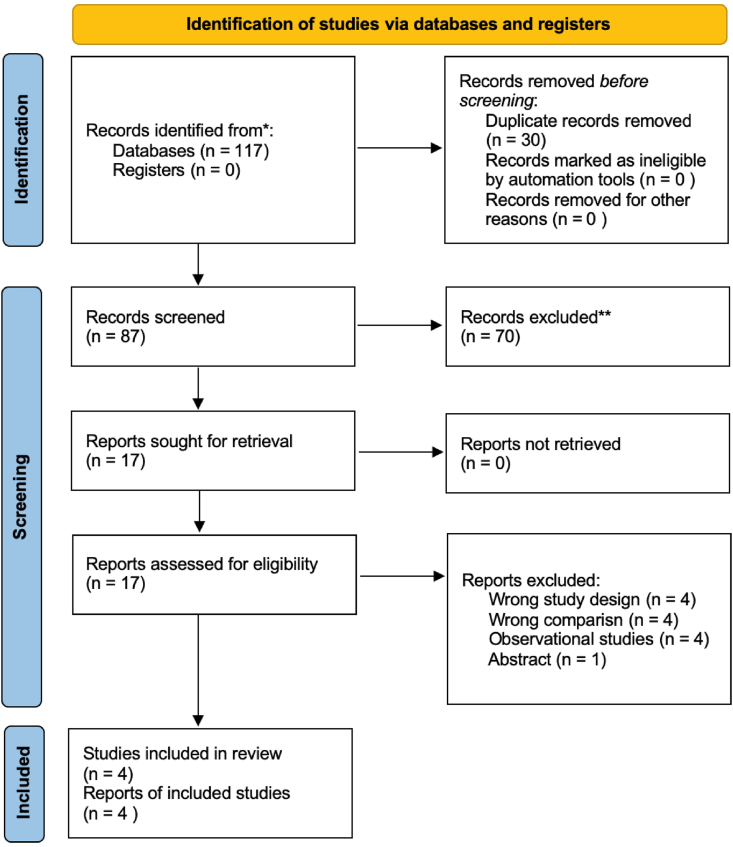
PRISMA flowchart in the search strategy.

**Fig. 2:**
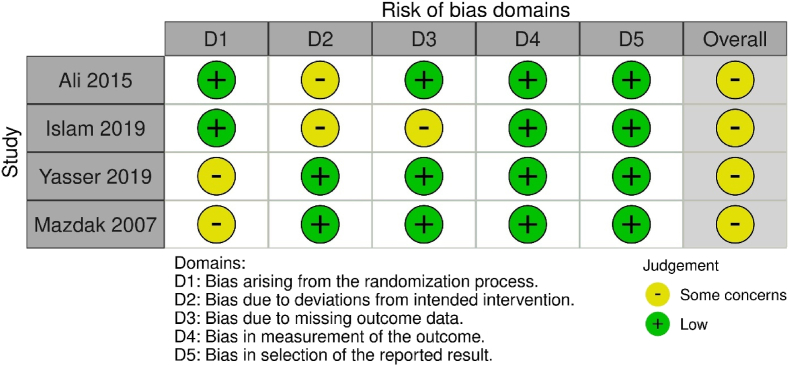
Risk of Bias Analysis using Cochrane Risk of Bias tools 2 for the evaluation of RCT.

The risk of bias in this study was carried out using the Risk of Bias tool 2 (RoB 2) developed by Cochrane (see Fig. 1). Our risk of bias assessment showed that all four included trials had some concerns in the overall result. These concerns observed in the trials were caused by the procedural aspect, and the type of surgery performed on patients cannot be completely confidential to the participants. Some data regarding the randomization process has also lacked a clear explanation. However, the result of the RoB in this study did not significantly impact the result of the analysis because the randomization process was still adequate. The summary for the overall RCTs risk of bias assessment was displayed in Fig. 2.

### 3.4 Grade evidence

The included studies of this analysis consisted of four RCTs focusing on the recurrence rate as the primary outcome. These studies presented no serious limitations, inconsistency, indirection, and imprecision due to adequate data and study design. The included studies are all considered high quality. Table 3 provided detailed information regarding the GRADE assessment.

**Table 3:** GRADE evidence profile: The efficacy and safety of mitomycin C intra urethral injection to prevent recurrent urethral stricture.

No of studies (Design)	Limitation	Inconsistency	Indirection	Imprecision	Odds Ratio (95%CI)	Quality
Recurrence rate 4 (RCT)	No serious limitation	No serious inconsistency	No serious indirectness	No serious imprecision	0.27 (0.16–0.45)	High

### 3.5 Recurrence rate

**Fig. 3:**
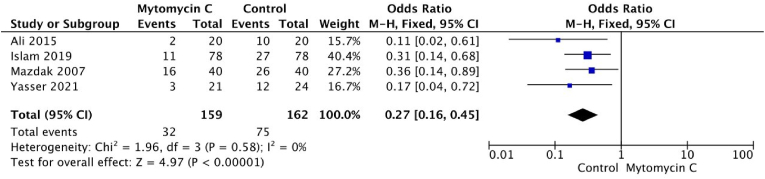
Forest plot analysis of recurrent urethral stricture between mytomicin C group compared to control group.

We managed to include four RCTs in the assessment of recurrence rate after the DVIU procedures, 159 patients were allocated to the group receiving additional treatment of mitomycin C, and 162 patients were allocated to the control group. The pooled analysis in the forest plot displayed in Fig. 3 showed a significantly lower recurrence rate in patients undergoing DVIU with additional treatment of mitomycin C compared to the control group (OR 0.27, 95% CI 0.16–0.45, p < 0.0001). The model for the analysis was carried out using a fixed-effects model due to the insignificant heterogeneity among the trials (P = 0.58, I^2^ = 0%).

## 4 Discussion

Urethral stricture is a formation of spongiofibrosis tissue in the anterior urethra. One of the mechanisms of action that have been proposed in the formation of urethral stricture is the presence of urine leakage in the corpus spongiosum tissue. This repeated process will result in a local inflammatory response in the corpus spongiosum tissue. In chronic circumstances, this process causes fibroblasts to initiate changes in the microenvironment, resulting in the creation of fibrotic tissue. The next stage of fibrotic tissue development is metaplasia of urethral epithelial cells into stratified squamous cells, which have a higher susceptibility to pressure changes. It has also been suggested that new tissue derived from stratified squamous cells is more vulnerable to damage and injury to the mucosa [23,24]. Several factors are reported to have a prognostic value for the incidence of recurrent urethral strictures, including, recurrent urinary tract infections, history of DVIU, long strictures (>2 cm), multiple strictures, strictures located in the bulbomembranous urethra, and extensive spongiofibrosis [25]. One of the most important factors that have an impact on this condition is that the instrumentation used in DVIU treatments can potentially injure healthy urethral tissue [25]. DVIU procedures are aimed to incise non-viable tissues. However, the problem arises as studies have shown that this procedure also causes trauma to the healthy urethral tissue and could increase the risk of extension of the stricture. Previous studies have also reported that the length of urethral stricture will increase after repeated DVIU procedures. In this study, we reviewed several RCTs that evaluated the efficacy of mitomycin C in reducing the recurrence rate after DVIU. An in vivo study by Kurt et al. that analyze the benefit of mitomycin C on mice showed a lower incidence of stricture recurrence in mice that received mitomycinC and triamcinolone compared to control. The author also reported that the number of fibroblasts and collagen was higher in the control group compared to mice receiving addition treatment [26]. In this review, we found four clinical trials that evaluated the role of mitomycinC in the incidence of stricture recurrence after DVIU procedures in humans. All the results from the trials showed a similar result. The incidence of stricture recurrence was lower in patients who received mitomycin C after DVIU procedures compared to the control group. Based on the meta-analysis result, we discovered that there was a significantly lower incidence of recurrent stricture in the group of patients receiving mitomycin C compared to the control group (OR 0.27, 95% CI 0.16–0.45, P < 0.0001). The pooled analysis showed that the heterogeneity among the included trials was low, and therefore the result of this meta-analysis had a high value of validity. One of the proposed mechanisms of action of mitomycin C in inducing repair of the urethral lumen after DVIU is by inhibiting DNA synthesis. DNA inhibition could reduce cell proliferation in the body, and therefore this agent could be beneficial in regulating uncontrolled cell proliferation. Several studies have shown the beneficial effects of mitomycin C in cancer treatment [27]. This effect was achieved due to the anti-proliferative properties of the agent that could suppress the growth of cancer cells. Similar to the cancer cells, injured urethral tissue also experiences proliferative imbalance. There is a presence of uncontrollable proliferating fibroblast cells in injured urethral tissues that cause the accumulation of type 1 collagen and promoting the formation of strictures. Several studies also reported increased fibroblast proliferation is associated with overexpression of extracellular matrix and Connective Tissue Growth Factor (CTGF) [28,29]. It will lead to the formation of abnormal urethral tissue, known as strictures [30]. The urethral stricture formation is characterized with complex mechanism and a tendency to form more fibrotic tissue after DVIU procedure. Giving mitomycin C to patients who had a simple urethral stricture undergoing DVIU was hypothesized to counteract the formation of new fibrotic tissues. Previous research has explained the effect of mitomycin C in inhibiting the proliferation of fibroblasts. This research also showed that there was a decrease in the viability of fibroblast cells after obtaining mitomycin C with a certain dose. Mitomycin C also has a dose escalation effect where the phenomenon of reducing cell viability will occur after an increase in mitomycin C dosage titration [31]. A recent study had shown that mRNA miR-21, a type of mRNA that influenced fibroblast cell division, had an association with the formation of fibrosis tissue. One pathway induced by miR-21 is the anti-apoptotic effect by inducing PDCD4 protein. Previous studies have explained that the inhibition in miR-21 will decrease in fibroblast cell viability [32]. Furthermore, the study implied there was an increase in Bax/BCL-2 protein in the results of western blotting fibroblast cells after getting miR-21 inhibitors. This condition induces fibroblast cell apoptosis pathways. The association between miR-21 and PDCD4 protein was also explained in this study, where PDCD4 protein would increase to other protein expressions called Rapamycin. The increase in Rapamycin protein will result in an exaggerated expromellullar due to the expression of cytokine IL-6 and IL-8. This study found that mitomycin C would significantly lower miR-21 and PDCD4 [33].

The result of this study provides evidence of the addition of mitomycin C in DVIU procedure. The incidence of stricture recurrence after DVIU could be reduced with mitomycin C. However, this study is not without limitations. In this study, we only address the analysis of the efficacy of mitomycin C in reducing the recurrence rate after DVIU, . Still, we cannot delve further to analyze the safety aspects of mitomycin C due to the data limitation. Future research should focus on the safety aspects of mitomycin C in DVIU. In addition, more objective parameters such as the scoring system to assess the symptoms of Lower Urinary Tract Symptoms (LUTS) such as IPSS, ICIQ-LUTS, and uroflowmetry could be used to get more assessment. Trials with a longer duration of follow-up are needed to assess the long-term effect of the mitomycin C. Finally, only four RCTs were included in the analysis of this study.Therefore, a further meta-analysis with more RCTs is needed to confirm the result.

## 5 Conclusion

Adjuvant treatment with mitomycin C improves the quality of the urethral lumen, reducing adhesions, and reducing the incidence of urethral stricture recurrence after DVIU procedures. Our findings highlight the benefits of adjuvant mitomycin C for reducing stricture recurrence in DVIU.

## Ethical approval

A systematic review does not require ethical approval. The protocol for this review has been registered in the PROSPERO database (CRD42021249209).

## Sources of funding

The authors received no financial support for the research.

## Author contribution

Concept – FHP; Design – FHP, FR, DMS; Supervision – FHP; Resources – FHP, FR, DMS; Materials – FHP, FR, FH, YPK, ZAR, DMS; Data Collection and/or Processing – FR, FH, YPK, ZAR, DMS; Analysis and/or Interpretation – FHP, FH, YPK, ZAR; Literature Search – FHP, FR, FH, YPK, ZAR, DMS; Writing Manuscript –FHP, FR, DMS; Critical Review – FHP, FR, DMS.

## Registration of research studies

Name of the registry: PROSPERO.

Unique Identifying number or registration ID: CRD42021249209.

Hyperlink to your specific registration (must be publicly accessible and will be checked): https://www.crd.york.ac.uk/prospero/display_record.php?RecordID=249209.

## Guarantor

Doddy Moesbadianto Soebadi.

## Informed consent

All involved participants have consented for their responses to be published anonymously.

## Consent

A systematic review does not require informed consent.

## Provenance and peer review

Not commissioned, externally peer-reviewed.

## Declaration of competing interest

No conflicts of interest in this paper.
